# Making sense of a cognitive behavioural therapy intervention for fear of falling: qualitative study of intervention development

**DOI:** 10.1186/1472-6963-14-436

**Published:** 2014-09-25

**Authors:** Tracy L Finch, Claire Bamford, Vincent Deary, Neil Sabin, Steve W Parry

**Affiliations:** Institute of Health and Society, Newcastle University, Baddiley-Clark Building, Richardson Road, Newcastle-upon-Tyne, NE2 4AX UK; School of Life Sciences, Northumbria University, Northumberland Building, Newcastle upon Tyne, NE1 8ST UK; STRIDE office, Newcastle University, 3/4 Claremont Road, Newcastle, Tyne and Wear, NE1 7RU UK; Institute for Ageing and Health, Newcastle University and Falls and Syncope Service, Royal Victoria Infirmary, Newcastle upon Tyne, NE1 4LP UK

**Keywords:** Fear of falling, Cognitive behavioural therapy, Falls, Ethnography, Process evaluation, Normalisation process theory, Intervention development

## Abstract

**Background:**

Fear of Falling (FoF) is commonly reported among older adults (up to 50%) and can impact negatively on physical and social activities, mood and quality of life. This paper explores the development, acceptability and feasibility of a cognitive behavioural therapy intervention (CBTi) for FoF.

**Methods:**

The process evaluation of the CBTi development phase of an RCT (conducted in the UK) reported here, used ethnographic methods. Data included: interviews with patients and carers (n = 16), clinic staff (n = 6) and the psychologists developing the CBTi (n = 3); observational field notes and transcripts of intervention development meetings (n = 9) and stakeholder engagement meetings (n = 2); and informal discussions with staff developing the CBTi (n = 8). Data collection and thematic analysis were guided by Normalisation Process Theory (NPT).

**Results:**

The process evaluation showed two domains of work necessary to develop a CBTi that made sense to stakeholders, and that could be delivered as part of an RCT. For the psychologists developing the content of the CBTi, a growing understanding of the complexity of FoF highlighted the need for an individualised rather than a manualised intervention. For the research team, the work involved adapting the structures and processes of the RCT to address preliminary concerns over the acceptability and feasibility of the proposed CBTi.

**Conclusions:**

Theory-based approaches to process evaluation can sensitise researchers to contested understandings about proposed interventions that could undermine implementation. Drawing on the coherence construct of NPT, this study emphasises the nature and extent of work required to ensure an intervention makes sufficient sense to key stakeholders in order to maximise chances of successful implementation.

**Electronic supplementary material:**

The online version of this article (doi:10.1186/1472-6963-14-436) contains supplementary material, which is available to authorized users.

## Background

As interventions to support the health and wellbeing of the population become ever more complex, increasing reliance is being placed upon applied social science for the production of knowledge that facilitates the translation of new treatments and interventions into everyday practice. The need for thorough understanding of the dynamics of complex interventions is recognised within the UK Medical Research Council’s framework for complex healthcare interventions [1] and the value of undertaking in-depth process evaluation of such interventions is both well understood and widely advocated [2]. This is reflected also in the recent explosion of empirical and theoretical literature focused on addressing well-documented problems concerning the uptake and routine embedding of evidence-based healthcare interventions, constituting an emerging field of applied research now known as ‘Implementation Science’. This paper describes how a Cognitive Behavioural Therapy (CBT) intervention for fear of falling in older people was developed to maximise its acceptability to intended clients and the likelihood of successful ‘normalisation’ into routine practice. The findings will be of relevance to the development of implementable complex healthcare interventions in other contexts.

The term ‘fear of falling’ (FoF) refers to a variety of psychosocial difficulties including fear, anxiety, loss of confidence, and impaired perception of ability to walk safely without falling [3, 4]. The syndrome is found in around 50% of community dwelling elders who fall, and up to 50% of those who have never fallen [3, 4]. Consequences include activity avoidance, social isolation and increasing frailty and risk of further falls independently of physical impairment [4, 5]. Although both common and debilitating, evidence of the effectiveness of interventions specifically targeting FoF remains limited and inconclusive [6]. There is some evidence supporting the use of physical and psychological therapies (in particular CBT) to improve the syndrome, although few studies have specifically targeted FoF as a primary outcome [3]. Whilst falls interventions have improved psychological outcomes in some studies [3], these benefits have not consistently been reported [7, 8]. Recent studies of CBT [9–11] have shown positive effects on FoF. So far, studies have used group-based CBT and therapy programmes have consisted of one or two sessions per week, over a duration of around eight weeks, with some advocating an additional ‘booster’ session in the medium term (e.g. six months [10]).

There is growing recognition of the diversity of older people with FoF and the simplistic traditional conceptualisation of FoF has been challenged [12]. Rather than being based solely in activity avoidance and deconditioning [9, 10], the causes and maintenance of FoF are likely to be multi-factorial [13]. CBT is thus likely to be beneficial in supporting this population [14], with adaptation to meet the needs of older people [15]. The cognitive behavioural model [16] of a problem situation being maintained by an interaction between physiological, behavioural, cognitive and affective responses is paradigmatic for FoF, and offers the hope of a viable therapeutic option. There is a need for many more trained cognitive behavioural therapists than are currently available; the development of a cognitive therapeutic package for the management of FoF that can be delivered routinely by non-specialist staff such as Health Care Assistants (HCAs) is vital if this common and debilitating condition is to be tackled effectively. CBT can be delivered by suitably trained non-psychotherapist staff [17, 18] but to our knowledge, this approach has not been attempted with HCAs in this context previously. In addition, only group interventions have been studied so far, with therapy delivered on a one-to-one basis yet to be tested in a FoF cognitive behavioural intervention study.

The development of effective interventions that can be sustained in routine clinical practice is facilitated by in-depth process evaluations to explore their feasibility and acceptability. Typically embedded within trials that aim primarily to address questions of clinical effectiveness, process evaluation is important not only for providing insight into the reasons why a trialled intervention has been shown to be clinically effective (or not), but it is also integral to understanding issues concerning transportability, workability, and integration of interventions into routine clinical practice. While process evaluations in FoF are limited, there is some evidence to suggest that group based CBT for FoF is feasible for participants and facilitators [19] and fits with regular care [20], though such data are based on questionnaire [19] and audit data [20] rather than a more robust framework to guide practice. In this study, we are drawing on Normalisation Process Theory [21], to explore psychological and sociological mechanisms of behaviour and action that have been empirically demonstrated to be important in the development, planning, and implementation of complex interventions [22]. By collecting longitudinal data using ethnographic methods (observation, interviews and informal discussions) we aim to gain an understanding of the social processes and relationships between all participants (patients, staff, researchers). This network of processes and relationships was the context in which the cognitive behavioural therapy intervention (CBTi) was to be developed and delivered. In seeking to understand the development of the CBTi, we consider the need for (i) the content of the CBTi to reflect the phenomenon of FoF, and (2) fit between the CBTi and the structures and processes of the RCT. In this paper we use data from the process evaluation of the intervention development phase of the study to explore how the systematic study of the acceptability, feasibility and development of a CBTi for FoF amongst multiple stakeholders both illuminated and informed the CBTi development process. Focusing on the ‘coherence’ aspect of NPT, we outline the challenges that occurred in this process and show how these were dealt with (or not) in the attempt to develop a psychological treatment for FoF that made sense to patients, their families, and healthcare providers.

## Methods

The process evaluation reported here is part of a study funded by the NIHR Health Technology Assessment programme [23]. The study includes two phases: (I) the development of a novel CBTi for FoF amongst community-dwelling older adults, and (II) a randomised controlled trial (RCT) to determine the effectiveness and cost-effectiveness of the CBTi amongst this population. The process evaluation spans both phases of the study, however the data reported here were collected in the intervention development phase of the project. The data comprises: (1) interviews with a sample of patients and carers/family members; (2) interviews and observation of professionals working in a community falls prevention clinic (CFPC); and (3) observation of intervention development meetings and related activities (e.g. briefing meeting for clinic staff; informal discussions with team members responsible for intervention development). The study methods are reported in adherence to the RATS guidance for reporting qualitative studies [24]. Before detailing these methods, a description of the intervention development methods is provided.

### CBTi development

Team members responsible for developing the CBTi were VD (a cognitive behavioural therapist and health psychologist with experience of developing and implementing and developing CBTis) and NS (a clinical psychologist experienced in working therapeutically with older people using CBT). The aim of this phase of the project was to develop a CBTi that could be delivered by HCAs (non-specialist, relatively low-paid staff), after training in basic CBT skills. The cognitive behavioural model [16] underpinning the intervention distinguishes between predisposing factors (what made a person vulnerable to a problem), precipitating factors (what triggered the current problem) and perpetuating factors (what is currently maintaining it). The model further distinguishes between physical, emotional, cognitive, behavioural and social factors in each domain. To develop the CBTi appropriately for the client group, assessment interviews with patients with FoF were undertaken by VD. The intervention development process also included the preparation of supporting materials (‘manuals’). An overview of the intervention using the TIDieR framework (template for intervention description and replication) [25] is presented in Table 1. A description of the content of each of the eight initial sessions and the six-month follow up session is provided in Table 2. Since treatment was based on an individual formulation, the detailed content of each session differed for each client.

**Table 1:** **Description of CBTi based on items included in the Template for Intervention Description and Replication (TIDieR) checklist**

	Item no item	
1.	**Brief name**	Stride
	Provide the name or a phrase that describes the intervention	
2.	**Why**	Based on the Cognitive Behavioural Model [16]
	Describe any rationale, theory, or goal of the elements essential to the intervention	
	**What**	
3.	**Materials**	Patient manual; therapist manual; training materials
	Describe any physical or informational materials used in the intervention, including those provided to participants or used in intervention delivery or in training of intervention providers. Provide information on where the materials can be accessed (such as online appendix, URL)	
4.	**Procedures**	Formulation, tailored intervention
	Describe each of the procedures, activities, and/or processes used in the intervention, including any enabling or support activities	
5.	**Who provided**	Health care assistants 5 day training programme
	For each category of intervention provider (such as psychologist, nursing assistant), describe their expertise, background, and any specific training given	
6.	**How**	Individual face-to-face sessions
	Describe the modes of delivery (such as face to face or by some other mechanism, such as internet or telephone) of the intervention and whether it was provided individually or in a group	
7.	**Where**	Patients’ homes (or convenient clinic if preferred)
	Describe the type(s) of location(s) where the intervention occurred, including any necessary infrastructure or relevant features	
8.	**When and How Much**	8 sessions over a period of 8 weeks each session lasting about one hour; homework to be completed between sessions. One review session six months after completion of initial intervention
	Describe the number of times the intervention was delivered and over what period of time including the number of sessions,their schedule, and their duration, intensity, or dose	
9.	**Tailoring**	Intervention to be determined by individual formulation
	If the intervention was planned to be personalised, titrated or adapted, then describe what, why, when, and how

**Table 2:** **Outline of CBTi sessions**

Session	Content
1	Assessment and formulation
2	Goals and target setting
3, 4, 6	Continuation
5	Review
7	Relapse prevention
8	Final review
Six month follow up	Review & recap, goals, setbacks, outcomes

### Process evaluation

#### Interviews with patients and carers

To assess the acceptability of the proposed CBTi, we recruited patients aged 60 years and over from a CFPC. Patients identified by their general practitioner as being at risk of falling are referred to this multidisciplinary clinic for comprehensive assessment of risk factors for falls. Consistent with criteria for the RCT (Falls Efficacy Scale- International (FES-I) [26] >23 [27]); no cognitive impairment (MMSE [28] > 23)^a^; and not already receiving a psychological intervention), patients were eligible for interview if they had significant FoF. Maximum variation sampling was undertaken by clinic staff, in terms of age, gender and FES-I score (patients’ scores on these measures were not available to the research team). Clinic staff briefly introduced the study to selected patients, who were invited to return an expression of interest form if willing to be contacted. Interested patients were then recruited and consented by research staff (CB). Where appropriate, carers and/or relatives of patients involved in the study were also invited for interview with the patient’s permission, and consented to the study. Participants were interviewed in their own homes. The sample consisted of 14 patients (aged 60–85, 9 female) and 2 carers (both female, age not specified). Interviews with patients were of 28–65 minutes duration, and with carers 29 and 34 minutes.

#### Interviews with professionals

The multi-disciplinary team working at the CFPC comprises a health care assistant, physiotherapist and a consultant geriatrician. These roles are covered by six individuals (three geriatricians, two physiotherapists and one health care assistant), all of whom were invited for interview, and took part (duration of interviews: 32–43 minutes). VD & NS were also formally interviewed using a semi-structured interview schedule (3 interviews, duration: 31–49 minutes).

All interviews conducted for this study were digitally recorded with participants’ consent. Interviews explored perceptions of key factors that might impede or facilitate the proposed CBTi being effective and workable in practice. The topic guides were loosely structured around Normalization Process Theory (NPT), but with ample scope for interviewees to respond openly about factors that they themselves saw as important to making the developing intervention effective and useful. The topics covered in the interview guides for patients and professionals are presented in Table 3.

**Table 3:** **List of topics in interview guides for patients and professionals**

	Patients	Professionals
Description of any falls and the immediate and longer term impacts of falling	✓	
Development of fear of falling and impacts on activities	✓	
Aims of the clinic and roles of professionals working within it		✓
Relevance of fear of falling to work at the clinic		✓
Barriers to addressing fear of falling at the clinic		✓
Feasibility of recruiting patients from the clinic		✓
***Current approaches to helping patients with fear of falling***		
Perceived value of different strategies for patients with concerns about falling (e.g. exercise, environmental adaptations, planning based, psychologically based)	✓	✓
***Proposed CBT intervention for fear of falling***		
Perceived value of cognitive behavioural therapy for fear of falling	✓	✓
Perceived acceptability of CBT to patients and barriers to participation	✓	✓
Evaluating the outcomes of CBT for fear of falling	✓	✓
Suggested name for CBT intervention	✓	✓

#### Observation

To gain an understanding of the clinical context of the study, each professional was observed at the CFPC (n = 5 clinical sessions). Professionals sought verbal consent from patients and any companions for the observation. No audio, or audio-visual recordings were undertaken of these observations.

The regular meetings of the CBTi development team (VD & NS) were audio recorded and observed by the researcher (CB) (n = 9 meetings, duration 27–51 minutes). These data were supplemented with fieldnotes taken of informal discussions (n = 8, duration 7–45 minutes) with VD outside these meetings. Other related stakeholder engagement activities, including a briefing meeting for clinic staff (duration 82 minutes) were also observed.

#### Data analysis

Audio recordings were transcribed, checked and anonymised. All data were analysed thematically using a constant-comparison technique [29]. This process allowed for the meaning of the data – and themes represented within it – to emerge freely without the constraints that might be imposed on the data if coding to a pre-specified coding frame. Themes were identified individually by CB and TF, who developed initial ideas and undertook coding independently, and then came together to discuss and compare ideas. We systematically worked through different data sources (professional interviews; patient interviews; intervention development meetings; interviews and informal discussions with the intervention development researchers; and fieldnotes) identifying themes separately for each type of data prior to producing an overarching coding frame. The involvement of the intervention development researchers in reviewing and commenting on the coding frame and subsequently on drafts of this paper provided an opportunity for respondent validation. There were no formal opportunities for respondent validation by patients and carers; however, the identification of similar themes in the interviews conducted by one of the intervention development researchers (VD) and those conducted as part of the process evaluation, provides some evidence of trustworthiness. Anonymised data (fieldnotes and transcripts) were imported into Nvivo to facilitate coding and data management. Data reported here are attributed using the unique identifier allocated to the participant. Prefixes are as follows: R denotes project team members; C denotes clinic staff; P denotes patients; F denotes family members.

The aim of the process evaluation for this study was not merely to evaluate the development of the CBTi, but also to collect data to inform and optimize the CBTi for delivery within the RCT. This was achieved through iterative data collection and feedback loops (that included formal and informal project-related meetings, and face-to-face and email communications with VD and NS and the wider project team), to enable practical resolution of problems as they arose. The impacts of the process evaluation activities on the development and delivery of the CBTi will be presented in the final section of the results.

### Approvals

The study was approved by NHS REC Newcastle and North Tyneside 1 (11/NE/0090). The study sponsor is Newcastle upon Tyne Hospitals NHS Foundation Trust.

## Results

The first section of the results explores how the intervention developers adapted the proposed CBTi to fit with their emerging understandings of FoF and achieved an intervention which, from their perspectives, made sense. The second section highlights the concerns expressed by a range of stakeholders over the acceptability and feasibility of the proposed CBTi and RCT, in particular the ‘fit’ of trial structures and processes with: older people; the staff who would be delivering the intervention; and the CPFC in which patients were to be recruited. We examine the work undertaken by the research team to improve the *coherence* of the trial to stakeholders.

### Achieving coherence within the CBTi

Based on their initial views that FoF could be conceptualised as anxiety-based and avoidant, the intervention developers had anticipated developing a simple, linear, manualised intervention which would centre around graded exposure. The initial clinical interviews with patients (to be reported elsewhere) revealed that FoF was a complex and multidimensional phenomenon with diverse precipitating, perpetuating and predisposing factors:

*“…I’m interviewing these people and I’m thinking ‘oh my God you are all so different’, how are we are going to translate this into an intervention?” (R4)*

The picture was further complicated by the range of psychosocial factors which also contributed:

*“It isn’t, or in very few is it focussed, like you might get focussed anxiety disorder, or fairly ‘clean’ depression as it were, this is much more illness, social stuff, everybody dying on you, swollen legs, pain, hips, there’s much more going on.” (Intervention development meeting, 19.10.2011)*

These observations created uncertainty about the feasibility of developing a CBTi suitable for all patients:

*R12 “…it may be that there are certain groups within that, those complex dimensions that you’ve already described, that actually this may not be an appropriate intervention with all […] groups.*

*R4 Aye, absolutely.*

*R12 … you’re already saying that this group [of patients with multiple co-morbidities] is a very complex one with…*

*R4 I’d be struggling to know what to do with them, to be honest. You can imagine a little change, but you’re up against the real, as it were.” (Intervention development meeting, 22.11.2011)*

Although some core themes began to emerge as the number of clinical interviews increased, the issue of how to manage the complexity of FoF remained. The intervention developers decided to centre the CBTi on individualised formulations thus enabling the intervention to be tailored to meet each patient’s individual circumstances and needs:

*R4 “the assessment should lead them to an individualised formulation, so say that you figure out OK this person is mainly phobic – so they’re having thoughts that ‘if I go out something terrible is going to happen’ so they’re avoiding. So the main intervention is going to be some form of gradual exposure, maybe getting them to walk out with a partner in the middle of the day in good weather, when they feel safe, building up on that partner walking. So it would be gradual exposure and some basic cognitive work, maybe, like a behavioural experiment: how much do you think this is going to be difficult, how was it, re-evaluate when you come back… so behavioural stuff plus basic cognitive stuff around that too. Something like that?*

*R12 Yes, absolutely.*

*R4 Yes, and with a more complex case which could be something like chronic pain plus dizziness plus diabetes plus hips (laughs) then what the hell are you going to do, is the question. But one that maybe targets… certainly all of it's going to be target setting in terms of realistic targets and then teaching people, maybe some acceptance, mindfulness stuff around the pain, doing some motivational stuff around actually what would get you out of the house, doing some basic challenging stuff around ‘OK your prediction is I’m too tired and too painful, but in fact from previous experience does it make you feel better when you go out’ – again, basic behavioural experiment: try going out. ‘Did you feel any worse than you thought you might?’ So, CBT for physical symptoms kind of approach.” (Intervention development meeting, 22.11.2011)*

The complexity of the intervention was often illustrated in discussions in intervention development meetings. For example, in the extract above whilst said jokingly, the comment *‘what the hell are you going to do?’* nevertheless highlights that delivering the intervention to some patients would be challenging, even to an experienced psychologist.

Broadening the focus of the CBTi to include work on pain, fatigue and other issues raised issues over the scope of the intervention and fit with the protocol:

*R1 so I think there’s a boundary issue about are we doing CBT for fear of falling,*

*R4 The boundary issue is key.*

*R1 Or are we doing CBT for fear of falling and actual (R4: and pain and ..)*

*[…]*

*R12 …in a sense you'll be working with them with whatever they bring, I mean it might be that you know the pain is a major factor in them not going out or whatever so I think that’s a legitimate piece of work. (Intervention development meeting 15.2.2012)*

In order for the intervention to be tailored to meet the constellation of specific issues faced by each individual, the intervention developers identified the range of skills that would be required:

- Ability to develop a therapeutic relationship
- Formulation skills
- Managing uncertainty during the process of learning CBT skills
- Willingness to practice new skills under scrutiny
- Ability to contain potentially distressing emotions
- Knowing own limitations and ability to judge when referral to more experienced professionals is required.

This created concerns over the potential mismatch between the complexity of the client group, level of experience of the therapists and the allotted time frame:

*“Because you’ve identified very challenging, difficult clients […] in terms of actual CBT, how do we get those relatively inexperienced therapists to actually be able to collect this information and actually then work – develop a relationship – and then work with these people over six sessions or however long it’s going to be. Because it’s such a small period of time.” (Intervention development meeting, 22.11.2011)*

At the same time there was an emphasis on being realistic and focusing on the aim of the project:

*“We’re not training psychological therapists; we’re training some healthcare assistants to deliver a very, very short intervention. So we’ve got to be clear about what’s possible as well.” (Intervention development meeting, 15.3.2012)*

Concerns were also raised about the potential generalizability of the intervention. There was an awareness that in light of the careful selection, training and supervision of health care assistants to meet the demands of this complex CBTi, the findings may not be replicable elsewhere:

*“All of which does raise some questions about generalisability, if we were recruiting the ones who are a bit savvy and we’re giving them a lot of training […] that doesn’t necessarily translate into your ‘Joe Bloggs care assistant’ who is already working in a very busy … but we’ll see, I think we have to prove it’s doable first.” (Intervention development meeting, 13.9.2011)*

### Achieving coherence in RCT structures and processes

The previous section focused on the work undertaken by the intervention developers to produce a CBTi that both met the (newly identified) needs of the client group and was deliverable by HCAs in the time available. However, the initial interviews with patients and clinic staff indicated a number of other issues which needed to be addressed to maximise the feasibility and acceptability of the RCT in which the CBTi was to be evaluated. These issues were iteratively fed back by staff responsible for the process evaluation (CB and TF) to key members of the project team to allow the development and implementation of strategies to address them prior to the start of the RCT.

### Perceived acceptability of CBTi to older people

Initial interviews with patients and clinic staff highlighted a number of issues over the perceived value of the proposed CBTi.

*“I imagine that some stoical north easterners might think that it was wishy washy mumbo jumbo that wasn’t necessarily going to help, you know the idea of going and sort of seeing like, effectively having a sort of a counselling type sessions, some people might not take that seriously.” (C2)*

A key issue raised by all stakeholders was the extent to which CBT would be acceptable to older people:

*F1 I don’t think it’s up P01’s street.*

*Int Right, well that’s really interesting, so why isn’t it, what do you think?*

*F1 Well, I just think he’s one of these, he likes to do his own thing and I don’t think he likes too much advice really, I mean he’ll listen to a bit of advice but whether he takes notice of it. I think the very words ‘cognitive behaviour’ and things, I think it’s just not up his street, you know. He doesn’t really analyse things, that’s my feeling. (F1)*

This family member also commented that the patient was ‘*not really a talker*’ and that ‘*I can’t see P01 dealing with his feelings, I really can’t*’. Similar themes of a reluctance to accept help were raised by another patient’s daughter:

*‘so I think although she would agree to any kind of help I think there’s a part of her that possibly feels she doesn’t need it, and I think it’s getting over that hurdle.’ (F2)*

Other participants, including P01 referred to above, acknowledged that an intervention for FoF might be useful for others, but that it ‘wouldn’t be for me’:

*I’m not sure I’m a very good candidate for you really; I think there are people much more in need of help than I am. My interests would just be general interest rather than need. (P09)*

A key issue concerned the name of the CBTi since concerns over terminology were consistently highlighted by patients and clinic staff. The use of terms such as ‘psychological’ and ‘psychotherapy’ were seen as potentially alienating to the intended client group:

*INT what does psychotherapy treatment mean to you?*

*P02 Sitting on a couch with a shrink!*

*INT And would you then sign up for that do you think?*

*P02 I don’t know, I don’t know whether that would help me with the problems I’ve got.*

It was seen as essential to ensure the title was positive; avoid the word ‘falling’ (as well as any words beginning with ‘psych’); and to consider including terms such as ‘confidence’ which people would be able to relate to. In addition to being non-threatening we wanted the name to convey a sense of the purpose and focus of the intervention so that it would not be mistaken for an exercise class. All stakeholder groups participating in qualitative interviews were asked for advice and suggestions; these were discussed in subsequent interviews:

*INT What about ‘a talking therapy to increase confidence’?*

*P03 […] No I don’t like that one because I think it’s got to be more than talking, you’ve got to be actually doing things haven’t you? (P03)*

Following discussions, we agreed on STRIDE as the brief study title (**Str**ategies for **i**ncreasing in**d**ependence, confidence and **e**nergy).

While there were clear reservations about the concept and name of the CBTi, stakeholders were generally positive about the practical aspects of the CBTi, for example, the number, frequency and duration of sessions, the timing of the follow-up session, and the intention to deliver the intervention at home. Patients emphasised the need for flexibility and for the sessions to fit around their other commitments. Several patients suggested group sessions either instead of or alongside individual sessions. Both advantages and disadvantages of group and individual sessions were identified, highlighting the importance of individual preferences:

*INT Ok, so why do you think one-to-one to begin with is a good idea?*

*P06 So that you know exactly what they want of you and what you’ve got to strive for, but once you’ve got that goal in mind and you’re managing to cope with it I think you could then join in with others.*

*INT And what would be the advantages of being in a group?*

*P06 Well meeting other people and maybe comparing how they’re getting on might be an idea.*

While the intervention developers discussed the feasibility of introducing group sessions in the light of patient comments, we found it difficult to reconcile the introduction of group sessions with the intention of the RCT to evaluate the effectiveness of individual CBT:

*R12 I think that a sense of group cohesion is an important one but it hasn’t got to be the main thrust of this treatment.*

*R4 Absolutely yeah.*

*R12 Well we don’t need to muddy the water* (*Intervention development meeting 15.2.2012)*

### Perceived value of CBTi for FoF

When asked to give their views about the proposed CBTi for FoF, clinic staff raised the possibility that, for some patients, FoF may actually be *protective* with regard to falling:

*“I don’t know whether it’s protective or not which would be my other question, is it a good thing people have a fear of falling, if you take that fear of falling away are they going to fall because their sense of caution has gone?” (C3)*

Clinic staff also expressed reservations over patients’ willingness to engage with an intervention that focused on FoF:

*“I’m not sure how receptive they will be to addressing fear of falling as a primary factor in the absence of anything else, any modifications to gait or aids or physical performance, so I don’t know how often it’s a stand-alone problem.” (C3)*

In general, staff expressed a preference for interventions which were consistent with their emphasis on identifying medical causes for falls; they were less convinced of the value of psychological interventions such as CBT:

*“So if you were saying to me they can have CBT or they can have an exercise group what would I choose? I would choose the exercise group because they have definite weakness and you can see benefits of them going to an exercise group and when you hear the feedback from people going to exercise groups it might be that I’m sure it has, it reduces their fear of falling and it might not but they are much more confident, they’re going outside, they’re going on walks, it’s really changed their lives.” (C4)*

Since the clinic staff would have a key role in ‘selling’ the CBTi to eligible patients in Phase 2 of the study, we organised a briefing meeting for clinic staff in which the intervention developers fed back the results of the interviews, described the intervention and illustrated how it would be relevant to different types of clients. This enabled staff to recognise the similarities between the CBTi and their existing practice:

*C3 well we use CBT quite a lot in chronic pain and back pain; it’s a fairly familiar thing and yes I’m sure it will be helpful for people.*

*C5 I mean I’ve used it a lot with working with older people and it’s not actually called CBT but it’s what you do with them from the point of view you’ve gradually got to build their confidence up through very simple increasing their activities and things and the way you talk to them and the way you encourage them and things like that as well, so yes I think it would be very, very helpful actually. (Briefing meeting for clinic staff)*

### Recruitment of patients to an RCT

Given the emphasis within the CFPC on routinized, structured assessments and medical interventions, the research team were concerned about the ‘room’ for a CBTi for FoF within this context:

*“The first thing that struck me when I went to the clinic is ‘where is the room?’ But what you are pointing out is not just the kind of the physical and temporal room but also a kind of emotional and habitual room.” (Intervention development meeting, 30.9.2011)*

Recruiting patients was therefore highlighted as a potential problem due to time pressures and the lack of any routine discussion of FoF within the CFPC. However, both team members who observed routine sessions at the CFPC felt that it would not be possible to ask staff to explore FoF in their consultations since - *‘the machine would collapse’* (Intervention development meeting, 30.9.2011). To facilitate recruitment in these difficult circumstances, we produced a user-friendly leaflet and a ‘script’ which staff could follow (Additional file 1). This would have minimal time implications and would follow their existing approach to referring patients for strength and balance classes provided by a local voluntary organisation.

The briefing meeting for staff (described above) also gave the research team a chance to explore and address staff uncertainties and anxieties about the implementation of the RCT:

*“Again fally, fraily people, you don’t want them left somewhere in a corridor falling over. I know it sounds pathetic but these kinds of things [you need to be] very, very careful of.” (C4, Briefing meeting for clinic staff)*

We were able to provide reassurance about the venue of the treatment (usually in patients’ own homes), and also to address other concerns, for example, mechanisms for informing patients’ GPs about their participation.

### Relevance and acceptability of proposed outcome measures

A final issue that emerged from discussions with clinic staff related to the adequacy of the FES-I as a means of identifying potential participants and the primary outcome measure despite clear published evidence of its validity [26]. These concerns over the outcome measure undermined the perceived validity of the proposed RCT. A key concern was the perceived lack of correlation between subjectively reported FoF and FES-I scores:

*“I don’t think the Tinetti […] really seems to correlate hugely with whether they are actually are afraid of falling.” (C2)*

Staff suggested two potential reasons for this apparent lack of correlation. First, their impression was that patients did not always answer the questions as intended, for example, dismissing them as irrelevant:

*she’s an agoraphobic and was very, very frightened of falling […]she even has the hairdresser coming to her house, she won’t go to the hairdresser and that sort of thing, but because the FES was so low from that point of view then obviously she wouldn’t fit your criteria then would she? (C5, Briefing meeting for clinic staff)*

Secondly, staff felt that responses often reflected functional limitations (e.g. arthritis or other pain) that affect mobility rather than FoF. Given the extent of clinic staff concerns over the use of the FES-I as the primary outcome measure, we added a visual analogue scale on which patients could rate their FoF, to be included alongside other measures to be used in the randomised controlled trial.

## Discussion

The aim of this paper was to explore the development, acceptability and feasibility of a CBT intervention for FoF for community-dwelling older people. The work described in this paper related to the intervention development phase of a larger study that includes a randomized controlled trial of the CBTi. In relation to this development phase, the embedded process evaluation has explored the CBTi development process from the perspectives of a range of stakeholders that included patients, carers, clinic staff working in the CFPC, and experienced psychologists tasked with developing the intervention.

The initial assessment interviews conducted to inform the intervention development opened up and challenged notions of the concept of ‘fear of falling’, uncovering both its heterogeneity (diversity of views and experiences) and complexity (in terms of associations between falls, appraisals of these, and related behaviours). In terms of heterogeneity, the differing nature of the ‘stories’ that are told in different spaces about a patient’s falling has been previously documented [30]. In developing the intervention the psychologists had to acknowledge and accommodate the importance of the network of co-morbid physical complaints, inter-personal relationships and/or social isolation revealed as central to FoF. This was addressed by introducing individualised formulations which added to the complexity of the intervention and created uncertainty over the feasibility of successful delivery of the CBTi by relatively inexperienced HCAs. The complexity of FoF highlighted here, reflects recent arguments for a deeper understanding of FoF that have developed out of analysis of associations of structured measures relating to the concept [13]. Our findings also support guidance for the adaptation of CBT for older populations, particularly the recommendation to avoid approaches relying on rigid manualisation [15].

Although not mentioned at all by patients, some professionals suggested that FoF could potentially be protective for avoiding falls; the CBTi carried the risk of making people ‘overconfident’ and thus increasing the risk of falls. It should be noted however that there is emerging evidence to suggest that FoF itself can impact negatively on a person’s mobility [31], thus increasing the likelihood of falling. We would also argue that FoF is debilitating for many, and that benefits experienced by individuals from reducing the negative impact of FoF on their confidence and participation in everyday activity should be a valued outcome independent of any reduction in falling [6].

In this paper, we have highlighted the work required to develop an intervention that would ‘make sense’ to key groups whose participation is expected and/or required. For the CBTi for FoF this included addressing key issues such as determining what to call it, developing participants’ understandings of what it would involve and what benefits it might have for patients, and anticipating the skills and training needs of the HCAs who were expected to deliver it. Our study has demonstrated that the development of an intervention that makes sense to its participants depends on exploring and understanding *where* and *how* the intervention might fit into the different contexts for which it is intended. Situated within a larger research study, the work undertaken here involved multiple contexts that needed to be considered at various points. This included the homes and lives of patients and their carers (since the intention was for the CBTi to be delivered in their homes, and to fit with their domestic schedules); the (relatively) bounded space of the intervention development process and the academic and professional expertise on CBT that framed this endeavour; the CFPC and its routines and personnel; the larger research study and data collection procedures associated with it; and the context of wider clinical practice (‘the real world’) into which the CBTi would hopefully be integrated. Tensions in meeting the requirements across these contexts were evident in several discussions. For example, the discovery of the degree of complexity of FoF challenged expectations that the CBTi could be delivered by non-specialist staff, which in turn threatened confidence that the CBTi would be implementable on a larger scale in ‘the real world’. This uncertainty over whether non-specialist staff could deliver the CBTi effectively could not ultimately be addressed through the CBTi development process but was acknowledged to be one of the fundamental research questions of the trial and the most relevant to the implementation of the CBTi into routine healthcare. Work also needed to be done to improve the fit between the study and the CFPC in terms of addressing uncertainty expressed by staff members about the relevance and value of the intervention (e.g. staff engagement activities), and adapting processes of enrolment of patients into the study. In our case, the CBTi was not to be delivered from within the CFPC setting, however the commitment and participation of clinic staff was essential to the conduct of the research study.

The process evaluation for this project is being framed by Normalization Process Theory (NPT) [21], and our interview schedules have included questions derived from the theory to guide our data collection and subsequent analysis. NPT is all about understanding the collaborative ‘work’ that needs to be done for a new intervention to become embedded within a given context. This gave us an appropriate lens through which to explore how an intervention (CBT) could be suitably developed for a particular patient/client group (those with FoF). During this first phase of the larger project, the main focus has centred around developing a shared sense of *coherence* regarding the CBTi. Here, we have demonstrated the importance of understanding, firstly, how people make sense of the problem (is there a problem, if so, for whom?) Secondly, we have shown how this can be used as a basis for developing an intervention that makes sense to those whose participation in it is necessary. As proposed by NPT, coherence work provides a necessary (though not sufficient) foundation for effective enrolment and commitment to participate (termed *cognitive participation*) [21]. As our process evaluation progresses through the RCT phase of the project, we will be able to draw more comprehensively on the other constructs of the NPT– cognitive participation (engagement and commitment), collective action (how people make the intervention work in action), and reflexive monitoring (how the work is appraised and reflected upon). We aim to provide insights to support or challenge these concepts in relation to embedding a CBTi for FoF in the context of a RCT, with an emphasis on translation of the CBTi into wider clinical practice. For other studies involving the development and evaluation of new healthcare interventions, the work presented here highlights the need for in-depth exploratory work *before* an RCT protocol is developed [1]. This is often done (e.g. stand-alone pilot/feasibility studies) but not always, and our study has shown that the assumptions of very experienced clinicians and academic researchers about not only a planned intervention, but the very nature of the clinical phenomenon the intervention is intended to address, may be challenged and require additional consideration and action in order to make the intervention ‘work’.

A potential limitation of this study is an underrepresentation of the perspective of family members, having only recruited two family carers to this phase of the project. At the outset, we had anticipated that the views of family members could potentially influence the uptake of CBT by older people, but in practice we found it difficult to recruit family members. This seemed to reflect the client group attending the clinic, who had relatively few health problems, usually attended the clinic alone, and were often very independent. For this population, the concept of family ‘carers’ was often irrelevant.

A key strength of this study is the depth of qualitative data collection and analysis that has been conducted, which has allowed the capture of important data concerning the processes (and challenges) of developing a CBTi in this context. The openness of the research team to a degree of observation and self-reflexivity exceeding that which would normally be expected in such studies has been the key to this. The study therefore provides a rich account of the process of developing a CBTi for a particular client group, from which other research projects may benefit. However, this paper has also demonstrated the value of undertaking in-depth process evaluation alongside the development and conduct of an RCT [32], in an iterative manner that allows for timely feedback to optimise processes where necessary.

## Conclusion

Our in-depth study of the development of a CBTi for FoF has shown that maximising the likelihood of interventions being both effective for their intended recipients and becoming a sustainable part of healthcare practice, depends on in-depth understanding of the diverse experiences, motivations and perspectives of key stakeholders. Theory-based approaches to intervention development can be useful for sensitising developers to contested understandings about healthcare problems and possible solutions that could undermine implementation if not addressed. Drawing on NPT, this study emphasises the nature and extent of work required to ensure an intervention has sufficient coherence to merit further evaluation.

## Endnote

^a^The FES-I and the MMSE were both routinely administered in the CFPC.

## Electronic supplementary material

Additional file 1: **Skills required to deliver the CBTi.** Draft script for introducing the study. (DOCX 14 KB)
