# Supplementary material for: Making sense of a cognitive behavioural therapy intervention for fear of falling: qualitative study of intervention development
Source: BMC Health Serv Res. 2014 Sep 25;14:436. doi: 10.1186/1472-6963-14-436 (PMC4263069; doi:10.1186/1472-6963-14-436)
Supplement: Supplementary file 1 — Additional file 1: Skills required to deliver the CBTi. Draft script for introducing the study. (DOCX 14 KB) [file 12913_2014_3552_MOESM1_ESM.docx]

**Additional File 1: Script for clinic staff introducing the STRIDE study**

We are currently offering patients the opportunity to take part in a study to look at practical ways to help them keep moving, increase their confidence and maintain their independence. It's aimed at people who have, for whatever reason, become a bit more cautious about their walking. It's called the STRIDE study. I've got information here about the study [give leaflet]. If you are interested in taking part, or would like to talk it over further, please fill in this form and give it back to me, or you can send it back in this envelope. This just lets the study team know you are interested, and they will then contact you and you can talk it over with them, and decide if you would like to take part.

[If the patient seems potentially interested, clinic staff can suggest completing the form there and then but will need to stress that sending back the form does not commit the patient to taking part, simply to finding out more about the study.]
